# Whole-body magnetic resonance imaging vs. positron emission tomography-computed tomography in pediatric oncology: enhancing diagnostic precision for solid tumors

**DOI:** 10.1590/1806-9282.20251462

**Published:** 2026-06-19

**Authors:** Süreyya Burcu Görkem, Gökalp Çıkman

**Affiliations:** 1 Erciyes University, School of Medicine, Department of Pediatric Radiology – Kayseri, Turkey.; 2 Adana City Training and Research Hospital, Pediatric Radiology Clinic – Adana, Turkey.; 3 University of Toronto, The Hospital for Sick Children, Department of Pediatric Neuroradiology – Toronto (ON), Canada.

**Keywords:** PET-CT, Diagnostic imaging, Comparative study, Child, Adolescent, Neoplasms, Whole body imaging

## Abstract

**OBJECTIVE:**

Pediatric solid tumors require accurate imaging for staging, treatment planning, and follow-up. Whole-body magnetic resonance imaging has emerged as a promising radiation-free alternative to positron emission tomography-computed tomography, offering superior soft tissue contrast. This study compares the diagnostic performance of whole-body magnetic resonance imaging and positron emission tomography-computed tomography in detecting primary tumors, metastases, and recurrences in pediatric solid tumors.

**METHODS:**

This retrospective study included 47 pediatric patients (27 boys and 20 girls; age range: 3–18 years; mean age: 11.3±3.5 years) with suspected or confirmed solid tumors who underwent both whole-body magnetic resonance imaging and positron emission tomography-computed tomography between 2017 and 2020. Lesional and per-patient agreement between modalities was evaluated using positron emission tomography-computed tomography as the reference. Statistical analysis was performed to assess inter-modality agreement.

**RESULTS:**

Positron emission tomography-computed tomography detected pathological findings in 35 patients (74.4%), including 27 primary tumors (57.4%), 18 metastases (38.2%), and one recurrence (2.1%). Whole-body magnetic resonance imaging showed high diagnostic concordance, detecting all primary tumors and the recurrence, and 16 of 18 metastatic cases. Per-lesion analysis revealed 66 lesions on positron emission tomography-computed tomography and 64 on whole-body magnetic resonance imaging, with two metastatic lesions (an ossified pulmonary metastasis and an osteoblastic femur lesion) missed by magnetic resonance imaging. Cohen's kappa (κ) for abnormality detection was 0.89, indicating almost perfect agreement (95%CI 0.75–1.00). McNemar's test showed no statistically significant difference between the modalities (p=0.479).

**CONCLUSION:**

Whole-body magnetic resonance imaging demonstrates excellent agreement with positron emission tomography-computed tomography for evaluating pediatric solid tumors, offering a radiation-free alternative, particularly advantageous in long-term follow-up. While limitations remain in detecting sclerotic metastases, whole-body magnetic resonance imaging is a primary alternative in specific scenarios.

## INTRODUCTION

Pediatric solid tumors are a heterogeneous group of malignancies that arise from various tissues and organs, including bone, soft tissue, and organs such as the liver, kidney, and brain. These tumors are distinct from adult cancers in terms of their histology, biology, and clinical behavior, necessitating a specialized approach to their diagnosis and management^1^. Accurate assessment of these tumors is critical for determining the appropriate therapeutic strategies and improving patient outcomes. Given the aggressive nature of many pediatric cancers and the risk of metastasis, comprehensive imaging plays a pivotal role in staging disease treatment, treatment planning, response monitoring, and long-term follow-up. Positron emission tomography combined with computed tomography (PET-CT) has been the cornerstone imaging modality for evaluating pediatric solid tumors. PET-CT provides valuable metabolic and anatomical information, facilitating the detection of primary tumors, metastatic spread, and treatment responses^2^. However, the cumulative radiation dose from repeated PET-CT scans during cancer management can further heighten the risk of secondary malignancies, emphasizing the need for safer imaging alternatives. WB-MRI offers superior soft tissue contrast, enabling detailed visualization of primary tumors, bone marrow metastases, and lymph node involvement without the use of ionizing radiation. It offers a valuable radiation-free imaging option in pediatric oncology, where minimizing cumulative radiation is critical. It is particularly effective in detecting and monitoring malignancies such as neuroblastoma, Ewing sarcoma, rhabdomyosarcoma, and lymphoma, supporting comprehensive assessment for staging, metastasis screening, and follow-up. However, limitations include longer acquisition times that may require sedation in younger patients, as well as higher costs and limited availability. Recent advancements in MRI technology, including faster sequences and improved whole-body imaging protocols, continue to enhance its practicality and integration into clinical practice^3^. The clinical significance of comparing WB-MRI to PET-CT lies in determining whether WB-MRI can match or surpass the diagnostic performance of PET-CT while mitigating radiation risks. By highlighting the advantages and limitations of both imaging techniques, this study aims to provide valuable insights into the evolving role of WB-MRI in pediatric oncology and its potential to become a standard imaging modality in the comprehensive care of children with solid tumors.

## METHODS

The institutional ethical board (Erciyes University School of Medicine, 2020/367; 07/08/2020) approved this retrospective study.

### Study design and patient selection

This retrospective study included 47 pediatric patients (27 boys and 20 girls; age range: 3–18 years; mean age: 11.3±3.5 years) with suspected or confirmed solid tumors who underwent both WB-MRI and PET-CT between 2017 and 2020 (Table 1). Patients were identified through a retrospective review of institutional imaging and oncology records. The decision to perform both WB-MRI and PET-CT was not based on a standardized institutional protocol but was determined at the discretion of the managing oncology team, depending on clinical factors such as diagnostic uncertainty, the need for comprehensive staging in high-risk or atypical tumors, or complex follow-up scenarios where additional imaging clarity was required. This non-random selection may introduce selection bias and is acknowledged as a limitation of the study. Inclusion criteria were as follows: (a) patients with histologically confirmed or clinically suspected primary solid tumors, (b) patients with known or suspected metastases or recurrence at the time of evaluation, (c) patients undergoing initial staging or follow-up evaluation who had both PET-CT and WB-MRI performed within a clinically relevant time window (≤2 weeks apart), (d) patients with no evidence of residual disease on follow-up imaging or post-surgical pathology. Our exclusion criterion was patients who underwent chemotherapy, radiotherapy, or surgical intervention after PET-CT but before WB-MRI (n=5), as such treatments could alter imaging findings and confound comparative assessment.

**Table 1:** Distribution of pediatric solid tumors in the study cohort (n=47).

Tumor	Number of patients
Ewing sarcoma	10
Neuroblastoma	9
Osteosarcoma	6
PNET	4
Rhabdomyosarcoma	4
Adrenocortical tumor	4
Synovial sarcoma	2
Ganglioneuroblastoma	1
Germ cell tumor	1
Hepatoblastoma	1
Immature teratoma	1
Infantile fibrosarcoma	1
Colon adenocarcinoma	1
Malignant melanoma	1
Askin's tumor	1

PNET, primitive neuroectodermal tumor.

### Positron emission tomography-computed tomography protocol

PET-CT was performed by the Philips Gemini TF 64 PET-CT device [tumor imaging procedure according to the European Society for Nuclear Medicine (EANM) guideline version 2.0.4]^4^. Patients’ oral intake was stopped at least 4 h before the 18F-FDG injection. In addition, blood glucose was checked before the extraction. In PET-CT, 5–7 MBq/kg of fluorodeoxyglucose was used intravenously as a radiopharmaceutical. Images were taken from the vertex of the head to the heels 60–80 min after the 18F-FDG injection using the 3D application. The total examination time in the PET-CT room was between 1.5 and 2 h.

### Whole-body magnetic resonance imaging protocol

All MR images were performed by a 1.5 T MRI unit (Siemens, MAGNETOM Aera, Germany). For each patient, from head to toe, coronal and axial or sagittal images were obtained in short tau inversion recovery (STIR) sequence [TR (ms)/TE (ms)=5,000/75; inversion time=160 ms to null fat sign; slice thickness=4–5 mm; slice gap value 1 mm; matrix value= 256×256, and field of view (FOV) coronal=350–400 mm and 200–400 mm, for axial images] without administration of any contrast agent by using standard head and neck coils, standard posterior spine coil, and 2× anterior body coils for thorax, abdomen, and limbs. Patients were told to use the toilet before the examination. Younger children who could not follow the instructions received sedation (intranasal 0.1–0.2 mg/kg, Dormicum, Roche). The patient's position was head-first with arms down by their side or crossed on their body. The total examination time in the MRI room was between 20 and 45 min.

### Image evaluation

Two pediatric radiologists (SBG and GC) jointly reviewed all WB-MRI examinations in a consensus reading session. The reviewers were blinded to patient clinical data and PET-CT findings to minimize interpretation bias. WB-MRI images were evaluated using a dedicated PACS workstation (Picture Archiving and Communication Systems, Sectra IDS7), and findings were subsequently compared with the corresponding PET-CT reports.

### Statistical analysis

All statistical analyses were performed using IBM SPSS Statistics version 22 (IBM SPSS Inc., Chicago, IL, USA). The distribution of continuous variables was assessed using the Kolmogorov-Smirnov test. To compare the diagnostic performance of the two imaging modalities, McNemar's test was employed for analyzing paired binary outcomes, such as differences in detection rates, reflecting whether the proportions of positive findings differed significantly between modalities. To evaluate the agreement between the two imaging modalities beyond chance, Cohen's kappa coefficient was calculated, providing a measure of inter-modality reliability. A p-value less than 0.05 was considered statistically significant for all tests.

## RESULTS

In total, 47 children diagnosed with solid tumors were assessed through a retrospective review. Among these, 35 patients (74.4%) demonstrated pathological findings on PET-CT, including 27 with primary tumors (57.4%), 18 with metastases (38.2%), and one case with postoperative recurrence (2.1%). The remaining 12 patients (25.5%) exhibited no pathological findings on PET-CT and WB-MRI at either initial diagnosis or during follow-up, indicating complete concordance. WB-MRI showed high diagnostic concordance, detecting all primary tumors and the recurrence, and 16 of 18 metastatic cases. Notably, 28 patients underwent whole-body MRI (WB-MRI) at the time of initial diagnosis and 19 patients received both initial and follow-up WB-MRI studies. Between two and nine WB-MRI examinations were performed per patient, with a maximum follow-up duration of 1 year. The overall kappa value (κ) for any abnormality detection was 0.89 (95%CI 0.75–1.00), which indicates almost perfect agreement (95.7%) between WB-MRI and PET-CT per patient. McNemar's yielded a p-value of 0.479, indicating no statistically significant difference in malignancy detection between the two imaging modalities per patient.

**Figure 1:**
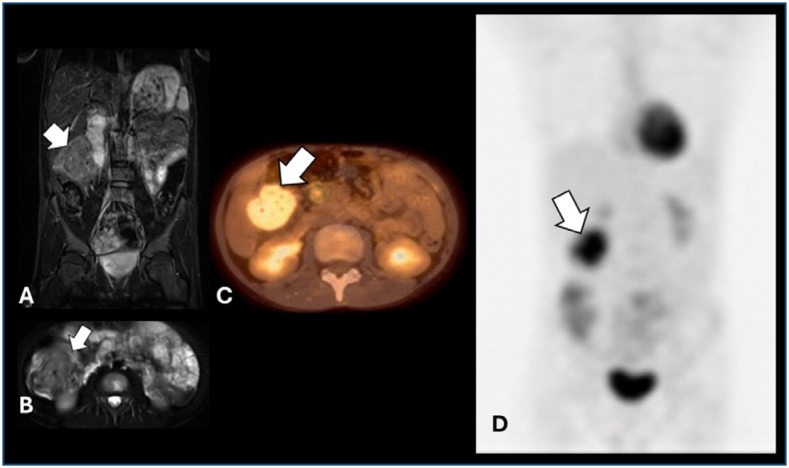
**(A–D)** 16-year-old boy with colon adenocarcinoma: coronal **(A)** and axial short tau inversion recovery images **(B)** of whole-body magnetic resonance imaging through the abdomen demonstrate heterogeneous hyperintense mass in the right hemicolon (white arrows). Positron emission tomography-computed tomography fusion axial image **(C)** indicates increased Fludeoxyglucose F 18 uptake (white arrow) in the mass. Positron emission tomography-computed tomography metabolic map image **(D)** shows Fludeoxyglucose F 18 uptake compared to surrounding tissues.

**Figure 2:**
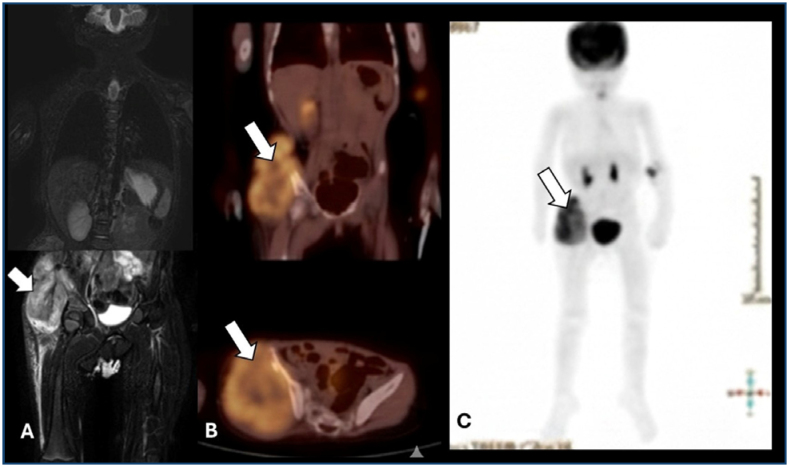
**(A–C)** 3-year-old girl infantile fibrosarcoma: coronal whole-body magnetic resonance imaging short tau inversion recovery image **(A)** demonstrates the right hemipelvic hyperintense solid mass (white arrow). Positron emission tomography-computed tomography fusion coronal and axial images show the right hemipelvic solid mass (white arrows) with an increased Fludeoxyglucose F 18 uptake **(B)**. Positron emission tomography-computed tomography metabolic map image **(C)** shows Fludeoxyglucose F 18 uptake compared to surrounding tissues (white arrow).

**Figure 3:**
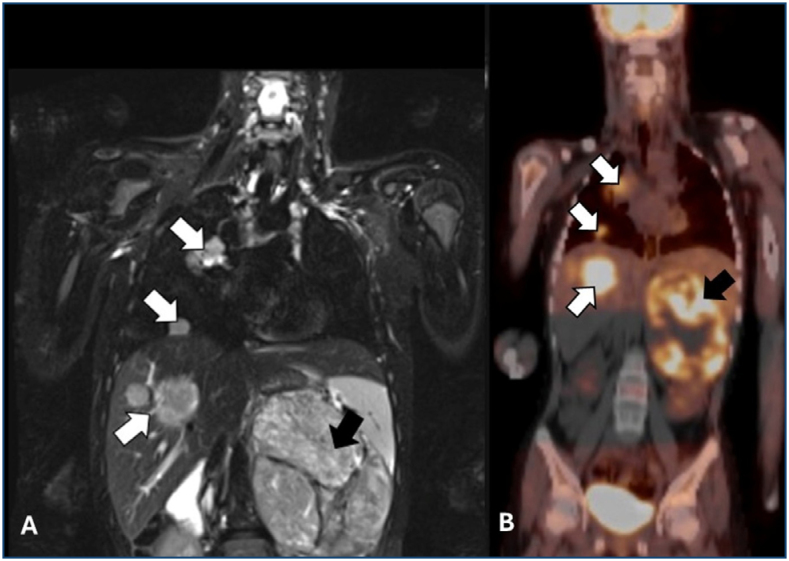
**(A, B)** 18-year-old girl with left adrenocortical carcinoma: Coronal whole-body magnetic resonance imaging short tau inversion recovery image **(A)** shows the hyperintense solid mass in the left adrenal region (black arrow) associated with multiple liver and lung metastases and hilar lymphadenopathies (white arrows). positron emission tomography-computed tomography image **(B)** demonstrates the same findings with increased Fludeoxyglucose F 18 uptakes (white arrows and black arrow).

**Figure 4:**
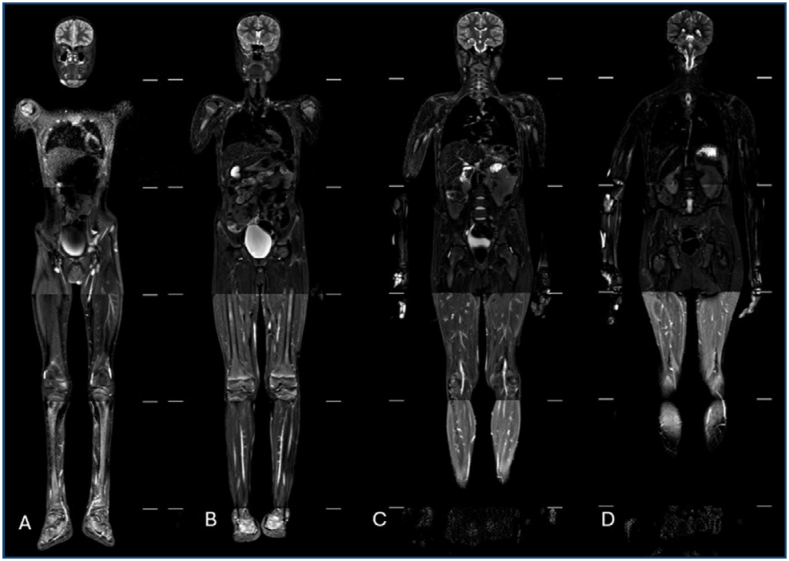
**(A–D)** 12-year-old boy with synovial sarcoma: consecutive coronal whole-body magnetic resonance imaging short tau inversion recovery images **(A–D)** through whole body show no metastasis or recurrence on follow-up.

On a per-lesion basis, a total of 66 lesions were detected on PET-CT, comprising 27 primary tumors (40.9%), 10 nodal metastases (15%) located in cervical, axillary, mediastinal, hilar, and abdominal regions; 12 bone or bone marrow metastases (18%); 5 peritoneal or retroperitoneal metastases (7.5%); 11 visceral metastases [7 pulmonary, 3 hepatic, and 1 central nervous system (16.6%)]; and 1 postoperative recurrence (1.5%). WB-MRI identified a nearly identical lesion distribution with a total of 64 lesions, including 27 primary tumors (42.1%), 10 nodal metastases (15.6%), 11 bone or bone marrow metastases (17%), 5 peritoneal/retroperitoneal metastases (7.8%), and 10 visceral metastases [6 lung, 3 liver, 1 central nervous system (15.6%)], as well as the single recurrence case (1.5%). WB-MRI failed to identify two lesions seen on PET-CT: an osteoblastic femur metastasis and an ossified lung metastasis. In each case, it resulted in a change in clinical staging from localized to metastatic disease, leading to escalation of chemotherapy intensity and inclusion of curative protocols such as localized surgical resection or focal radiotherapy. A comprehensive summary of patient-level imaging agreement is presented in Table 2. Comparative examples illustrating modality-specific findings and representative cases are shown in Figures 1A–D, 2A–C, 3A, B, and 4A–D.

**Table 2:** Lesion-based comparison of positron emission tomography-computed tomography and whole-body magnetic resonance imaging findings in pediatric solid tumors.

Imaging findings	PET-CT (n, %)	WB-MRI (n, %)	Agreement (%)	p-value (McNemar's)
Primary tumor	27 (40.9%)	27 (42.6%)	100%	N/A
Lymphadenopathy	10 (15%)	10 (15.6%)	100%	N/A
Bone/bone marrow metastasis	12 (18%)	11 (17%)	91.6%	*p>0.05*
Peritoneal/retroperitoneal metastasis	5 (7.5%)	5 (7.8%)	100%	N/A
Visceral metastasis	11 (16.6%)	10 (15.6%)	90.9%	*p>0.05*
Postoperative recurrence	1 (1.5%)	1 (1.5%)	100%	N/A
All findings (total)	66 (100%)	64 (100%)	96.9%	0.479

Note: Agreement values are based on per-lesion analysis. Percentages reflect the proportion of each lesion type relative to total PET-CT and WB-MRI detections. McNemar's test p-values>0.05 indicate no statistically significant difference in detection rates between modalities. PET-CT: positron emission tomography-computed tomography; WB-MRI: whole-body magnetic resonance imaging.

## DISCUSSION

This study highlights the potential of WB-MRI as a valuable imaging modality in the evaluation of pediatric solid tumors. By retrospectively comparing WB-MRI findings to those of PET-CT—a widely used reference in oncologic images, we assessed the diagnostic concordance between the two modalities in detecting primary tumors, metastases, and recurrences. Our findings demonstrated a high degree of agreement between WB-MRI and PET-CT (κ=0.89, 95%CI 0.75–1.00) when PET-CT was used as the comparator. These figures indicate that WB-MRI often matches other modalities, but they show agreement between methods, not accuracy. This near-equivalent performance is particularly noteworthy given the absence of ionizing radiation in WB-MRI, which is a significant consideration in pediatric populations vulnerable to cumulative radiation risks. Given that many pediatric oncology protocols require repeated imaging, WB-MRI offers a compelling alternative in longitudinal care strategies^5^. Moreover, the ability of WB-MRI to detect the majority of metastatic lesions underscores its robustness and supports further evaluation in prospective, protocol-optimized settings.

Currently, standard imaging procedures for neuroblastomas and sarcomas involve a combination of CT, MRI, I-123 MIBG scintigraphy, and PET-CT, depending on the patient's specific needs^6^. Our findings suggest that WB-MRI could potentially replace or complement these modalities in selected cases, especially when reducing radiation burden is a clinical priority. Nevertheless, a multidisciplinary imaging approach remains essential, and WB-MRI should be considered as part of a broader diagnostic framework rather than a standalone replacement^7^.

WB-MRI also plays a valuable role in monitoring tumor response during neoadjuvant chemotherapy or radiotherapy, offering precise anatomical detail that can support surgical planning and guide ongoing treatment decisions. Due to its high soft tissue resolution and lack of ionizing radiation, WB-MRI is particularly well-suited for repeated use in pediatric patients. Although PET-CT remains essential for assessing metabolic activity and identifying certain metastatic patterns, its effectiveness in evaluating treatment response is limited to changes in metabolic uptake, which may not always correlate with structural tumor changes. In contrast, WB-MRI enables direct visualization of tumor size, extent, and tissue characteristics, making it a strong candidate for incorporation into both diagnostic and follow-up protocols, especially when minimizing radiation exposure is a priority. Rather than serving as a replacement, WB-MRI should be viewed as a complementary tool to PET-CT, with advantages in preoperative assessment, treatment monitoring, and long-term surveillance in pediatric oncology patients^8,9^. WB-MRI demonstrates considerable clinical value in the assessment of specific pediatric tumor types, including neuroblastoma, Ewing sarcoma, rhabdomyosarcoma, and lymphoma. It allows for comprehensive anatomical evaluation, including detection and characterization of primary tumors, assessment of bone marrow involvement, and monitoring of treatment response over time. The STIR sequence is central to WB-MRI, providing effective fat suppression and enhancing lesion visibility in bone marrow and soft tissues^10-12^. The two metastatic lesions missed by WB-MRI—an osteoblastic femoral metastasis and an ossified pulmonary nodule—reflect inherent limitations in MRI physics and common whole-body sequences such as STIR. These sequences are sensitive to lesions with high water content but are less effective in detecting dense, calcified, or sclerotic tissue. Osteoblastic metastases have low proton density and short T2 relaxation times due to their high mineral content, resulting in minimal signal on both STIR and conventional MR images. Similarly, calcified lung nodules contain little mobile hydrogen and often appear hypointense or are completely inapparent on MRI. In STIR sequences, fat suppression can further obscure such lesions, particularly when they are small or embedded within low-signal structures like bone or lung parenchyma. These technical constraints explain the false-negative findings in our study and highlight the need for complementary imaging approaches when such metastatic patterns are suspected. PET-CT provides superior sensitivity in detecting certain types of metastases, most notably small pulmonary nodules, which may be difficult to visualize on MRI due to motion artifacts and limited lung parenchyma contrast. Additionally, PET-CT enables quantitative metabolic assessment through standardized uptake values (SUV), which can be instrumental in evaluating treatment response, monitoring minimal residual disease, and guiding biopsy targets. PET-CT examinations also typically have shorter acquisition times and often do not require sedation, making them more practical in certain clinical settings, particularly for younger or uncooperative children^4^. In contrast, WB-MRI's strength lies in its high soft tissue resolution, spinal and bone marrow evaluation, and radiation-free follow-up potential—factors that are particularly valuable in pediatric patients requiring serial imaging. Given these complementary advantages, the two modalities may be best viewed as tools with overlapping and distinct roles. Future clinical algorithms could benefit from individualized modality selection based on tumor type, disease distribution, patient age, and specific diagnostic goals.

Currently, no universal imaging protocol exists for pediatric solid tumors, but the addition of sequences such as diffusion-weighted imaging (DWI) and T1-weighted imaging enhances WB-MRI's diagnostic performance, especially for identifying bone marrow and lymph node abnormalities^13,14^. Advanced techniques like perfusion imaging can further support evaluation of tissue vascularity and viability. Nevertheless, challenges remain—particularly the need for longer acquisition times and potential sedation in younger children, which can limit its practicality in some settings.

Recent studies suggest that streamlined WB-MRI protocols using rapid 3D STIR sequences may reduce scan time, improve patient compliance, and minimize the need for sedation without sacrificing diagnostic quality^10^. While our findings and existing literature support WB-MRI's utility, especially in radiation-sensitive pediatric populations, its reduced sensitivity for detecting certain calcified or ossified metastases remains a limitation. This highlights the importance of continued refinement of WB-MRI protocols and reinforces the role of PET-CT as a complementary modality in providing a more complete diagnostic assessment.

### Future directions and proposed whole-body magnetic resonance imaging protocol

Given the high concordance observed in this study, future research should aim to refine and standardize WB-MRI protocols to enhance diagnostic accuracy and clinical applicability. One key area of improvement lies in the integration of advanced sequences that are now widely used in modern WB-MRI practices. A proposed optimized protocol would include (1) coronal and sagittal STIR sequences for broad soft tissue and bone marrow screening; (2) axial DWI with background suppression, such as DWIBS (diffusion-weighted whole-body imaging with background body signal suppression), to improve sensitivity for small or early lesions; and (3) axial T1-weighted sequences for detailed anatomical localization and marrow characterization. The addition of contrast-enhanced sequences may be considered in selected cases where vascularity or lesion perfusion is critical, although contrast-free protocols retain advantages in safety, efficiency, and repeatability for longitudinal follow-up. Standardizing such a protocol across institutions would improve comparability, reduce variability, and better position WB-MRI as a primary imaging modality in pediatric oncology. Future prospective studies using this comprehensive protocol, ideally with histopathologic or long-term follow-up confirmation, are essential to validate and expand upon the findings of this retrospective analysis.

### Limitations

Despite its strengths, this study has certain limitations. The retrospective design introduces potential selection bias, as the choice to perform both WB-MRI and PET-CT was based on clinical discretion rather than a standardized protocol. Additionally, the absence of an independent reference standard, such as histopathology or long-term clinical follow-up, limits the ability to calculate true diagnostic accuracy. The lack of such a reference standard limits our ability to make definitive claims regarding the absolute diagnostic accuracy of either modality. The patient population was also heterogeneous in terms of tumor types and imaging timing (initial vs. follow-up), which may affect the generalizability of the findings. From a technical standpoint, WB-MRI failed to detect two specific ossified metastases due to limitations of standard MR sequences such as STIR, which is less sensitive to ossified low-proton-density lesions. Furthermore, we used only STIR sequences without DWI or contrast-enhanced sequences, which limits the generalizability of the results, as modern WB-MRI protocols often incorporate DWI to enhance sensitivity for small or subtle lesions and gadolinium contrast to improve lesion conspicuity and tissue characterization. Practical challenges also remain, including long scan times, the need for sedation in younger children, and limited availability in some healthcare settings.

## CONCLUSION

WB-MRI demonstrated high concordance and comparable diagnostic performance with PET-CT, avoiding radiation exposure in the evaluation of primary tumors, metastatic disease, and recurrence in pediatric solid tumors. Its ability to provide comprehensive, radiation-free whole-body imaging makes it a valuable modality in pediatric oncology, particularly where long-term surveillance is required. Although limitations exist—particularly in detecting sclerotic or calcified lesions—WB-MRI remains effective when appropriate sequences such as STIR and DWI are utilized.

Within the context of modern pediatric oncology practice, WB-MRI offers a complementary approach to existing imaging strategies. It enhances soft tissue assessment and supports safer longitudinal imaging by reducing cumulative radiation exposure. With ongoing technological advancements and expanding availability, WB-MRI is poised to become an increasingly vital component of staging, treatment planning, and longitudinal follow-up in pediatric patients with solid tumors. Prospective studies are essential to further validate its utility and refine its clinical indications.

## Data Availability

The datasets generated and/or analyzed during the current study are available from the corresponding author upon reasonable request.
